# Editorial: Plastic to Bioplastic (P2BP): A Green Technology for Circular Bioeconomy

**DOI:** 10.3389/fmicb.2022.851045

**Published:** 2022-04-18

**Authors:** Richa Priyadarshini, Thava Palanisami, Arulazhagan Pugazhendi, Arumugam Gnanamani, Obulisamy Parthiba Karthikeyan

**Affiliations:** ^1^School of Natural Science, Shiv Nadar University, Greater Noida, India; ^2^Global Innovative Centre for Advanced Nanomaterials (GICAN), The University of New Castle, Callaghan, NSW, Australia; ^3^Centre of Excellence in Environmental Studies, King Abdulaziz University, Jeddah, Saudi Arabia; ^4^Department of Marine Biology, Faculty of Marine Sciences, King Abdulaziz University, Jeddah, Saudi Arabia; ^5^Division of Microbiology, Central Leather Research Institute-CSIR, Chennai, India; ^6^Department of Engineering, College of Technology, University of Houston, Houston, TX, United States; ^7^Department of Civil and Environmental Engineering, South Dakota School of Mines and Technology, Rapid City, SD, United States

**Keywords:** plastic, circular economy, bioplastic, microbial degradation, PETase, recycling

The demand for fossil-derived plastic (FD-Plastic) has grown exponentially by 300% between 1999 and 2019; current production is ~400 million tons per year, of which only 9% recycled with the rest ending up either in landfill or the natural environment. It is expected that FD-Plastic production will further increase to about 600 million tons in 2025 and is projected to release 50 times higher carbon (e.g., 56 billion tons of CO_2_ eq.) than of all the combined coal power stations in the USA by 2050 (Geyer et al., 2017; Hamilton et al., 2019; Justine et al., 2019; Zaman and Newman, 2021). Several features such as high durability, corrosion and chemical resistance, lightweight, and low cost have made FD-Plastic a favorable material for industrial and household use. They undergo degradation through combined physical, chemical, or biological processes leading to release of microplastics, which enter the food chain and are biomagnified. The microplastics usually have different physio-chemical properties from that of FD-Plastic, whose interaction with the environment is complex to study and understand. The World Health Organization urged scientists to find a route to reduce FD-plastics' pollution and replace it with biodegradable plastics (Bio-Plastic) to reduce the environmental burden and human exposure (WHO, 2019).

In this special issue, we have proposed a model of developing “hybrid-plastic” i.e., blended Bio- and FD-Plastics and/or complete valorization of FD-Plastic as Bio-Plastic as an approach to re-incorporate those end-of-life FD-Plastics into the value chain. Microbes produce Bio-Plastic, such as Polhydroxyalkanoate (PHA) or polylactic acid (PLA), from sugars (derived from agricultural biomass such as sugarcane juice or corn or lignocellulosic biomass derived after pre-treatment) and/or from greenhouse gases [such as methane (CH_4_), carbon dioxide (CO_2_), etc.,] under fermentation conditions. However, the Bio-Plastic production was a meager 2.42 million tons (0.6% of FD-Plastic) in 2021 and is forecasted to reach 7.59 million tons by 2026 (European Bioplastic, 2021). Considering the future demands for FD-plastics and the problem associated with its disposal, it is very important to develop sustainable biotechnologies to convert the FD-Plastics' waste into value products. This requires a fundamental understanding of the interaction of microbes with the FD-Plastics, surface modification and pre-treatment for improved access for microbial attack, specific bioactive enzymes required for the mineralization, the environmental conditions for efficient conversion, and processing engineering approaches.

Specifically, the use of bioplastics in different areas has gained immense interest due to their biodegradable nature and environmental sustainability. Also, converting FD-Plastics to Bio-plastics (P2BP) has gained momentum in recent years. In this research topic, we compiled five different articles. Amobyone et al. provided a comprehensive review on microplastics and nanoplastics (MNP) in the environment. Specifically, the authors elaborate on MNPs transport into the marine ecosystem and their eventual accumulation in food chains, leading to various toxicological effects. On the other hand, microbes play an important role in FD-plastic biodegradation in the environment and accumulation of MNPs were summarized. A number of microbial species were isolated and characterized for the degradation of plastics including polyethylene terephthalate (PET). Qi et al. investigated the role of a synthetic microbial consortium for degradation of PET. The synthetic microbiome was developed using *Rhodococcus jostii, Pseudomonas putida*, and two metabolically engineered *Bacillus subtilis* that are known PET degraders. The bioengineered *B. subtilis* produced the enzymes PET hydrolase (PETase) and monohydroxyethyl terephthalate hydrolase (MHETase) and could degrade 13.6% of PET films in 7 days. In the case of the four-species consortium, the weight loss of PET film increased to 23.2%, demonstrating the potential of artificial microbial consortia in the biodegradation of complex polymers. This will leave the MNPs and some fossil carbons stored in biomass as bioplastics or other storage molecules. Samadhiya et al. discussed the bioplastic production potential of different bacterial and algal species using waste carbon sources. Most of the bioplastics accumulated by bacteria/algae were PLA or PHAs. The PHAs are biopolyesters produced by bacteria under nutritional stress. PHAs are biodegradable, non-toxic, and are considered a sustainable source for bioplastics. Vuong et al. conducted a global phylogenetic and ecological distribution study of potential PHA-producing Bacteria and Archaea. Genome mining was carried out for PHA synthase (PhaC), a key enzyme involved in the PHA biosynthesis, and these genome mining efforts uncovered previously unknown candidate taxa for PHA production. The main conclusion of this study was that PhaCs, in particular Class III genotypes, are more prevalent and diverse in composition within both Bacteria and Archaea. This *in silico* study could lay the foundation for future research into isolating and characterizing unique PHA-producing bacteria from the environment and exploit them for P2BP productions. Mahato et al. explored the use of different edible oils for the production of PHAs in *Pseudomonas aeruginosa*. Groundnut oil is rich in saturated fatty acids and was found to be the best carbon source for PHA production.

Nevertheless, the industrial scale production of Bio-Plastic and implementation of P2BP remains challenged by long-standing fundamental issues: (a) greenhouse gas emissions associated with the production of Bio-Plastic, production capacities by individual strains, and techno-economics of the process; (b) robust microbial strains, understanding of its FD-Plastic degradation pathways, and genetic engineering approaches to convert P2BP; and (c) developing an industrial scale microbial P2BP conversion process to meet supply and demand. We would like to acknowledge that significant progress has been made in isolating/characterizing the FD-Plastic degrading microbes and enzymes, as well as new advances in developing synthetic biology approaches and microbiome community developments for reducing the environmental burden.

The papers published in this research topic show the possibility of biodegradation of FD-Plastic, while no studies directly addressed the P2BP concepts. However, coupling of FD-Plastic degradation with Bio-Plastic production will be a feasible approach that requires more fundamental studies to advance research in this area (Figure 1). Specifically, future research should consider developing a robust consortium that simultaneously degrades FD-plastics and accumulates Bio-Plastic and optimizes the conditions for commercial use.

**Figure 1:**
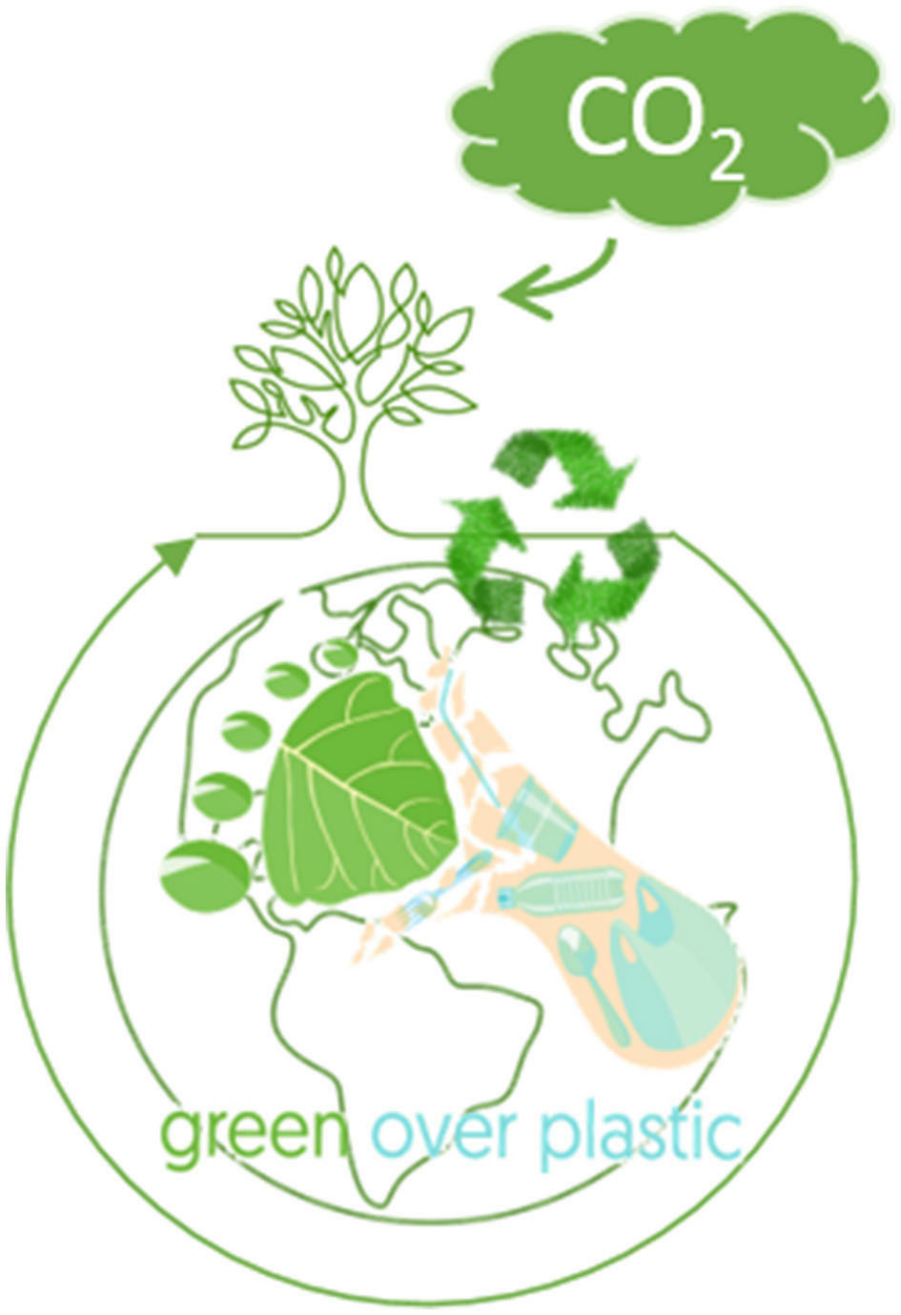
Green manufacturing of Bio-Plastics and recycling of FD-Plastics within the circular Bioeconomy.

## Author Contributions

RP and OPK wrote this editorial note. TP, AP, and AG edited the final text. All co-authors approved this final version.

## Conflict of Interest

The authors declare that the research was conducted in the absence of any commercial or financial relationships that could be construed as a potential conflict of interest.

## Publisher's Note

All claims expressed in this article are solely those of the authors and do not necessarily represent those of their affiliated organizations, or those of the publisher, the editors and the reviewers. Any product that may be evaluated in this article, or claim that may be made by its manufacturer, is not guaranteed or endorsed by the publisher.
